# Dispersion of Nanoparticles in Different Media Importantly Determines the Composition of Their Protein Corona

**DOI:** 10.1371/journal.pone.0169552

**Published:** 2017-01-04

**Authors:** Klemen Strojan, Adrijana Leonardi, Vladimir B. Bregar, Igor Križaj, Jurij Svete, Mojca Pavlin

**Affiliations:** 1 Group for nano and biotechnological applications, Faculty of Electrical Engineering, University of Ljubljana, Ljubljana, Slovenia; 2 Department of Molecular and Biomedical Sciences, Jožef Stefan Institute, Ljubljana, Slovenia; 3 Department of Chemistry and Biochemistry, Faculty of Chemistry and Chemical Technology, University of Ljubljana, Ljubljana, Slovenia; 4 Department of Organic Chemistry, Faculty of Chemistry and Chemical Technology, University of Ljubljana, Ljubljana, Slovenia; 5 Institute of Biophysics, Faculty of Medicine, University of Ljubljana, Ljubljana, Slovenia; Brandeis University, UNITED STATES

## Abstract

Protein corona of nanoparticles (NPs), which forms when these particles come in to contact with protein-containing fluids, is considered as an overlooked factor in nanomedicine. Through numerous studies it has been becoming increasingly evident that it importantly dictates the interaction of NPs with their surroundings. Several factors that determine the compositions of NPs protein corona have been identified in recent years, but one has remained largely ignored—the composition of media used for dispersion of NPs. Here, we determined the effect of dispersion media on the composition of protein corona of polyacrylic acid-coated cobalt ferrite NPs (PAA NPs) and silica NPs. Our results confirmed some of the basic premises such as NPs type-dependent specificity of the protein corona. But more importantly, we demonstrated the effect of the dispersion media on the protein corona composition. The differences between constituents of the media used for dispersion of NPs, such as divalent ions and macromolecules were responsible for the differences in protein corona composition formed in the presence of fetal bovine serum (FBS). Our results suggest that the protein corona composition is a complex function of the constituents present in the media used for dispersion of NPs. Regardless of the dispersion media and FBS concentration, majority of proteins from either PAA NPs or silica NPs coronas were involved in the process of transport and hemostasis. Interestingly, corona of silica NPs contained three complement system related proteins: complement factor H, complement C3 and complement C4 while PAA NPs bound only one immune system related protein, α-2-glycoprotein. Importantly, relative abundance of complement C3 protein in corona of silica NPs was increased when NPs were dispersed in NaCl, which further implies the relevance of dispersion media used to prepare NPs.

## Introduction

Despite numerous advances of nanotechnology in the area of biomedicine in recent years, the technology itself did not completely fulfil high expectations in the field of medical applications [1]. Although early NPs-based formulations like Doxil^®^ were very successful [2], some of the later nanomedicine products were not so triumphant and others were even retracted from the market (e.g. Endorem^®^ and Sinerem^®^) [3]. The domain of nanomedicine is wide and so are the reasons for the drawback. Problems with reproducibility and scaling up are sometimes tightly connected with insufficient characterization of nanomedicine formulations. To understand the effects of nanomedicines in vitro or in vivo, we need to consider several physico-chemical parameters, such as size, size distribution, surface area, charge and surface chemistry which forms synthetic identity [4]. The second level of characterization is performed when nanomedicines interact with biological systems [5]. This biological identity is defined also by adsorption of biomolecules, mainly proteins, to the surface of nanoparticles (NPs) and it is generally referred to as their protein corona [6–10]. Interaction between NPs and biological environment such as tissue and cells is mediated by this outmost layer of NPs, one of the frequently overlooked factor in the nanomedicine [11].

Significance of protein corona was demonstrated by numerous studies on different types of NPs and on various cell lines [12–22]. Already before NPs interaction with cells or tissue, proteins can affect the aggregation of NPs [12,13]. State of aggregation can determine the cellular uptake of NPs, which may be therefore directly connected to the composition of the protein corona [14–16]. As already demonstrated, the uptake of golden NPs with different coatings is a function of the composition of their protein coronas [17]. Also, the well-known poly(ethylene glycol) stealth effect responsible for reduced cellular uptake of NPs was recently explained by the presence of specific proteins in their protein corona rather than the absence of all proteins [18]. Too high cellular uptake of NPs can lead to cytotoxicity which is again known to correlate with the presence or the absence of their protein corona [19,22]. Interestingly, the presence of a single type of protein on the surface of NPs is enough to trigger immune response on the cellular level [20,21].

Many factors dictate protein corona composition of NPs and consequently their biomedical applicability. Factors governing protein corona composition of NPs can be divided to the ones defined by the NPs themselves [23–28] and others, outlined by the media where corona is formed [9,29–36]. Firstly, protein corona is NPs-specific—different materials bind different proteins [23]. Size and surface coating of NPs with identical composition are also relevant [23–26,28] and there was also an attempt to use protein pre-coating of NPs as a factor for controlling their protein corona composition [27]. Although relevant, factors defined by NPs are fewer compared to media-dependent factors. Composition of the NPs corona depends on the source of proteins [29–31], time [9,32] and temperature [33] of the incubation of NPs in biological fluids. Not all variables are so intuitive; corona composition can also be affected by serum heat inactivation [34], choice of anticoagulant [30], static versus dynamic conditions of the media [35] and even the presence of magnetic field in the case of iron oxide NPs [36]. Although many factors that define NPs protein corona composition are already well studied, there is one that has been addressed insufficiently—the composition of media used for dispersion of NPs.

There are at least two different media relevant when considering NPs protein corona formation: the first is the medium in which NPs are dispersed (i.e. medium used to dilute NPs), and the second is the medium where protein corona forms (i.e. medium used to dilute the source of proteins). Importance of the latter was clearly demonstrated by Maiorano et al. [37], but the effects of the former remains underestimated.

The main question addressed in the present work was, whether different dispersion media used for preparation of NPs affects the resulting protein corona or not. To answer this question, we have chosen two examples of nanoparticles: i) polyacrylic acid (PAA) coated cobalt ferrite NPs (PAA NPs), stable under physiological conditions, developed in our group for further use in biotechnology and biomedicine [38] and commercially available silica NPs, used in cleaning products for everyday use. Since there is a multitude of different nanoparticle systems and since the interaction with the dispersion media is very much dependent also on the properties of the NPs itself, we have selected these two NPs suspensions as an example of the potential media effects on protein corona composition.

Dispersions of NPs were prepared in four different biologically relevant media: Dulbecco`s phosphate buffered saline with CaCl_2_ and MgCl_2_ (PBS), 0.9% (m/v) NaCl, ATCC modified RPMI-1640 cell culture media (RPMI) and distilled water (dH_2_O). Protein corona was formed in 10% or 100% fetal bovine serum (FBS). Our results demonstrated that the medium in which NPs were dispersed had significantly affected NPs protein corona composition and could have an important implication on potential biological effects of NPs Moreover, there was a clear difference in protein corona composition between dispersion of PAA NPs and silica NPs in complex media where macromolecular corona was formed (e.g. RPMI) compared to media without macromolecules (e.g. dH2O).

## Materials and Methods

### Nanoparticles

Silica NPs were acquired from Nanotesla Institute Ljubljana (Ljubljana, Slovenia). Silica NPs were dispersed in a solution of citric acid in ethanol at pH 2. Z-average size of silica NPs in dispersion was 58.6 nm with polydispersity index (PDI) 0.46. PAA NPs were prepared as described elsewhere [38]. Briefly, cobalt ferrite (Co ferrite) NPs were prepared by co-precipitation [39] and stabilized in water. Alkaline medium was removed and NPs were re-suspended in distilled water. The process was repeated three times and after the last washing step nitric acid was added to stabilize NPs in acidic media. NPs were coated in situ with a 45% (m/m) water solution of polyacrylic acid sodium salt with molecular mass of 8 kDa (Sigma-Aldrich, St. Luis, Missouri, USA), by mixing 10 ml of ferro fluid and 10 ml of PAA water solution of equal mass concentrations at 20 mg/ml for 10 min at room temperature. Unbound polyacrylic acid was removed with dialysis against dH_2_O. dH_2_O was replaced every four hours, four times in total, to ensure removal of unbound polyacrylic acid. Larger aggregates were removed by filtration with Minisart^®^ filter unit (Sartorius Stedim Biotech, Göttingen, Germany), the size of pores was 0.20 μm. Z-average size of PAA NPs in dispersion was 116.2 nm with PDI 0.18.

### Characterization of NPs

ATR-FTIR spectra of dry samples were recorded on a Bruker FTIR (Fourier Transform Infrared Spectroscopy) Alpha Platinum ATR spectrophotometer (Bruker, Billerica, Massachusetts, USA). Samples were dried on to the surface of the ATR diamond crystal prior to measurements. Dynamic light scattering was measured using Malvern Zetasizer NanoZS (Malvern Industries, Malvern, UK) with the non-invasive backscatter algorithm. Z-average size, polydispersity index (PDI), and hydrodynamic diameter based on number distribution of particles are reported. Zeta potential was also measured on Zetasizer NanoZS, with disposable folded capillary cells and the M3-PALS measurements technology. The measurement was conducted after 5 min of stirring of colloidal suspension in the sample cell. The refractive index of 1.10 was used.

### Corona preparation

FBS (Sigma-Aldrich, St. Luis, Missouri, USA) was used to prepare protein corona of NPs. FBS was used as supplied stock solution (100% FBS) or diluted ten times (10% FBS) in PBS without CaCl_2_ or MgCl_2_ (Sigma-Aldrich, St. Luis, Missouri, USA). LoBind micro-centrifuge tubes (Eppendorf, Hamburg, Germany) were used for all experiments. Prior to corona preparation, we dispersed NPs to a final concentration of 1 mg/ml in the four analysed media: PBS (Sigma-Aldrich, St. Luis, Missouri, USA), 0.9% (m/v) NaCl (B Braun, Melsungen, Germany), RPMI (Gibco Laboratories, Gaithersburg, Maryland, USA) or dH_2_O. The pH of dispersion media was held constant for all experiments. Properties of dispersion media are reported in Table 1. Dispersions were vortexed and incubated for 5 min at room temperature. Afterwards, 100 μl of NPs dispersion (containing 100 μg of NPs) was added to 1 ml of 100% or 10% FBS, vortexed and incubated for 1h at 37°C. FBS-NPs mixtures were transferred to a new microcentrifuge tube and centrifuged at 15000 × g for 20 min at 4°C. Supernatants were removed and pellets dispersed in 1 ml of cold PBS without CaCl_2_ and MgCl_2_. This procedure was repeated three times. Proteins that remained adhered to NPs were considered as their protein corona [40]. Control samples were prepared in identical way with equal volume of dispersion media added instead of NPs.

**Table 1:** Characteristics of the media used to disperse PAA NPs and silica NPs.

Dispersion media	pH	Ionic strength [mM]	Osmotic concentration [mOsm/L]	Conductivity^b^ [mS/cm]
dH2O	7.0 ± 0.4	0.0	0.0	0.1
PBS	7.3 ± 0.3	166.0	287–309^a^	15.6
NaCl	7.0 ± 1.1	154.0	308	15.1
RPMI	7.2 ± 0.2	NA	246–306^a^	12.5

^a^ Reported by the manufacturer

^b^ Measured on Zetasizer NanoZS

### Sodium dodecyl sulphate polyacrylic gel electrophoresis (SDS-PAGE)

After the last centrifugation we removed supernatants and added 100 μl of non-reducing sodium dodecyl sulphate (SDS) sample buffer (10% (m/v) SDS, 25% (v/v) glycerol, 0.5% (m/v) Bromophenol Blue, 300 Mm TRIS-HCl, pH 8.8). Pellet was dispersed by vortexing and heating for 5 min at 95°C. NP-protein complexes were thus shattered and NPs were removed by centrifugation at 15000 × g for 20 min. The proteins that remained associated with NPs were considered as components of protein corona. Pierce^™^ 660 nm protein assay (Pierce Biotechnology, Rockford, Illinois, USA) was used for total protein quantitation. The absorbance of the particle-free and serum-free control was subtracted from the samples and total protein concentration was calculated relative to the bovine serum albumin standard. Aliquots of 20 μl were loaded on 10% (m/v) SDS-polyacrylamide gel in SDS running buffer (10 g SDS, 30.3 g TRIS, 144 g glycine in 10 l of dH_2_O) and resolved at 195 V. After electrophoresis, the gels were stained with AgNO_3_ and developed using Na_2_CO_3_.

### Mass spectrometry

Protein identification was performed by mass spectrometry (MS) analysis [25]. Selected protein bands, found exclusively in samples containing NPs, were manually excised from the SDS-PAGE gel. Spots were dissected to smaller pieces and de-stained with 1:1 volume ratio of 30 mM potassium ferricyanide and 100 mM sodium thiosulfate solution. Gel pieces were incubated in 200 mM NH_4_HCO_3_ solution for 20 min, washed two times with LC-MS Ultra CHROMASOLV^®^ water (Sigma-Aldrich, St. Luis, Missouri, USA), and dehydrated by covering with 100% acetonitrile. Reduction of proteins was performed with 10 mM dithiothreitol solution at 56°C for 45 min and alkylated by 55 mM iodoacetamide at room temperature for 30 min. Alkylation was stopped by addition of 25 mM NH_4_HCO_3_. Proteins were digested in gel with the MS grade modified trypsin (Sigma-Aldrich, Sigma-Aldrich, St. Luis, Missouri, USA) in 25 mM NH_4_HCO_3_ at 37°C overnight. Resulting peptides were extracted with 50% (v/v) acetonitrile / 5% (v/v) formic acid and concentrated in vacuum to 10 μL. Extracts were purified on StageTips C18 (Thermo Fisher Scientific Inc., Waltham, Massachusetts, USA) according to the manufacturer's instructions and analysed using an electrospray ionization (ESI) ion trap-MS (MSD Trap XCT Plus, Agilent, USA) as described in Leonardi et al. [41].

### Data analysis

DLS measurements were performed by twenty consecutive runs on individual sample, 30 seconds each. Zeta potential was measured by automatically determined number of consecutive runs. Z-average size, PDI, hydrodynamic diameter based on number distribution of particles and zeta potential with standard deviation are reported. Two sided Student’s t test with 0.95 confidence level was used to test for statistical significance. Liquid chromatography-ESI-MS spectral data, obtained as Mascot generic files (mgf), were analysed in the SwissProt database using the in-house Mascot search engine with following parameters: two miss cleavages were allowed, peptide and fragment mass tolerance of ±1.2 and ±0.6 Da, carboxyamidomethylcysteine (C) as fixed modification and oxidized methionine as variable. The results were validated using Scaffold 2 software (Proteome Software, Portland, Oregon, USA). Two sided Student’s t test with 0.95 confidence level with Hochberg-Benjamini correction was used to compare protein composition of samples prepared in PBS, NaCl and RPMI-1640 (separately for 10% and 100% FBS) to samples prepared in dH2O (separately for 10% and 100% FBS). Samples obtained from silica NPs and PAA NPs were analysed separately. Spectral counts matching to a protein are an indicator of its amount in a given sample [42]. Spectral counts were used to calculate relative protein abundance in each sample using the following formula:

$$RPA_{k}=\;\frac{SC_{k}}{\sum_{i=1}^{N}SC_{i}}\times 100\;,$$ (1)

where RPA_k_ is the relative abundance of the protein “k”, SC_k_ is spectrum count for protein “k” and $\sum_{\text{i}=1}^{\text{N}}{\text{SC}}_{\text{i}}$ is the sum of spectral counts in sample containing “N” proteins [17]. The sum of relative protein abundances of all proteins over a given sample is 100%.

Gene ontology (GO) annotation was acquired from UniProt knowledge database. Molecular masses and theoretical isoelectric points (pI) of proteins were calculated using Compute pI/Mw tool from the ExPASy portal. Data analysis was performed in R software environment (version 3.2.2). Experiments were done in three independent replicates if not stated otherwise.

## Results

### Characterization of NPs

**Fig 1:**
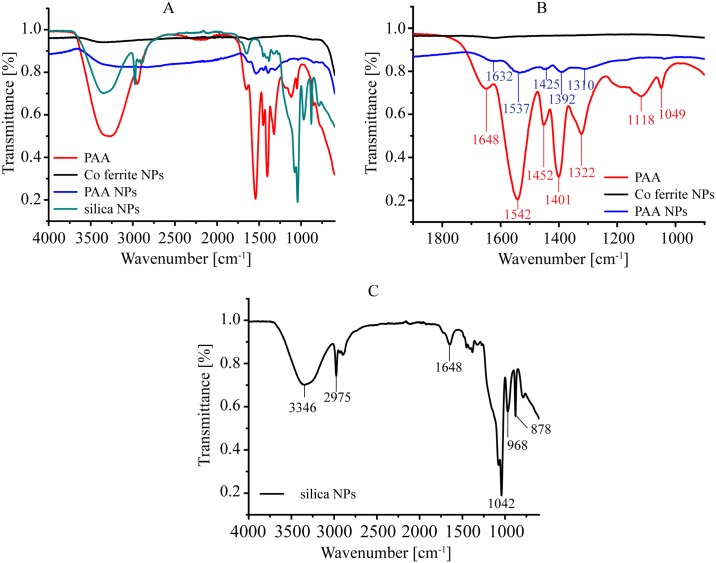
FTIR measurements. Samples were dried on the diamond crystal from solutions/dispersions prior to ATR-FTIR measurements. A: ATR-FTIR spectra of cobalt ferrite NPs (Co ferrite NPs), PAA, PAA NPs and silica NPs. B: ATR-FTIR spectra of cobalt ferrite NPs (Co ferrite NPs), PAA, and PAA NPs in the 1900–900 cm^-1^ region. C: ATR-FTIR spectrum of silica NPs in the 4000–600 cm^-1^ region.

PAA NPs with the Z-average diameter of 116.2 nm and zeta potential of -59 ± 4 mV (in dH_2_O) were prepared in dH_2_O and dried on to the surface of the ATR diamond crystal prior to ATR-FTIR measurements [43]. ATR-FTIR spectra of cobalt ferrite NPs (Co ferrite NPs) PAA, PAA NPs, and silica NPs measured between 600 and 4000 cm^-1^ are shown in Fig 1A. ATR-FTIR spectra of Co ferrite NPs, PAA and PAA NPs confirmed successful coating of cobalt ferrite with polyacrylic acid (Fig 1B). In ATR-FTIR spectrum of PAA (Fig 1B, red line), the absorption bands at 1648, 1542, and 1401 cm^-1^ (Fig 1B, red line) are shifted to 1632, 1537, and 1392 cm^-1^ upon coating to cobalt ferrite NPs (Fig 1B, blue line). This indicates binding of the deprotonated form of PAA to the NPs, which is additionally confirmed by the difference in wave numbers of other characteristic absorptions (Fig 1B) [43]. Silica NPs had the Z-average size of 58.6 nm and were originally stabilised with citric acid and dispersed in ethanol. Although dried, FTIR spectrum (Fig 1C) showed some residual ethanol with the peak at 3346 cm^-1^ (O-H stretching) and the peak at 2975 cm^-1^ (C-H stretching). Citric acid residue was responsible for peaks at 1648 cm^-1^ (C = O stretching) and also at 3346 cm-1 (O-H stretching), while peaks at 1042 cm^-1^ (Si-O-Si stretching), 968 cm^-1^ (Si-OH stretching), and 878 cm^-1^ (Si-O bending) confirmed the presence of silica.

In Table 2 we can see that there was no significant differences in size of PAA NPs between dispersion media (PBS, RPMI, NaCl), the Z-average size of PAA NPs was scattered around 180.4 nm (± 55.7 nm). The formation of protein corona in 10% FBS had not significantly changed the Z-average size of the PAA NPs. On the other hand, NaCl, PBS and RPMI reduced zeta potential from -59 ± 4 mV in dH_2_O to an average of -29 ± 2 mV in the other three media. Additional protein coating formed in 10% FBS further reduced zeta potential to an average of -16 ± 3 mV, regardless of the dispersion media. The Z-average size of silica NPs was higher in all four dispersion media compared to Z-average diameter of silica NPs dispersed in ethanol. High Z-average size and PDI values indicated agglomeration of silica NPs in all four dispersion media used. Moreover, zeta potential values are in agreement with this observation. Measured Z-average sizes in 10% FBS were lower for all dispersion media except for distilled water. Absolute zeta potential values were higher when measured in 10% FBS compared to measurements taken in media without FBS. It should be noted that PDI values were quite high (> 0.2), indicating instability of NPs dispersed in different media. This made size values very hard to interpret.

**Table 2:** Characterization of PAA NPs and silica NPs in different dispersion media with or without 10% FBS. Z-average size, hydrodynamic diameter, PDI values, and zeta potential are presented. Statistically significant differences determined for different media compared to samples in dH2O are denoted with an asterisk.

	Dispersion media	Z-average size (nm)	Hydrodynamic diameter (nm)^c^	PDI	Zeta potential ±SD (mV)
**PAA NPs**	dH20	116.2	55.5	0.18	-59 ± 4^a^
	PBS	206.4	50.4	0.69	-27 ± 1*
	NaCl	242.9	75.6	0.53	-31 ± 2*
	RPMI	156.1	42.4	0.72	-27 ± 2*
	dH20 + 10% FBS	167.4	77.0	0.19	-17 ± 3^b^
	PBS + 10% FBS	237.6	35.5	0.88	-16 ± 4*
	NaCl + 10% FBS	249.1	49.9	0.49	-16 ± 3*
	RPMI + 10% FBS	191.5	55.6	0.59	-14 ± 3*
**silica NPs**	ethanol	58.6	22.0	0.46	NA
	dH20	660.6	190.0	0.76	5 ± 9
	PBS	1556.0	141.0	1.00	-4 ± 0
	NaCl	2556.0	712.0	0.86	-1 ± 0
	RPMI	1619.0	295.0	1.00	-3 ± 0
	dH20 + 10% FBS	1489.0	10.0	0.99	-7 ± 0
	PBS + 10% FBS	32.0	6.5	0.33	-10 ± 0
	NaCl + 10% FBS	677.7	7.5	0.71	-9 ± 0
	RPMI + 10% FBS	286.9	6.5	0.49	-7 ± 0
	10% FBS^d^	88.8	8.7	0.17	-7 ± 0

^a,b^ Samples used for statistical comparison

* Statistical significance determined by two-sided Student’s t test (p<0.05)

^c^ Based on the particle number distribution

^d^ Without NPs

### Analysis of NPs protein corona with SDS-PAGE

**Fig 2:**
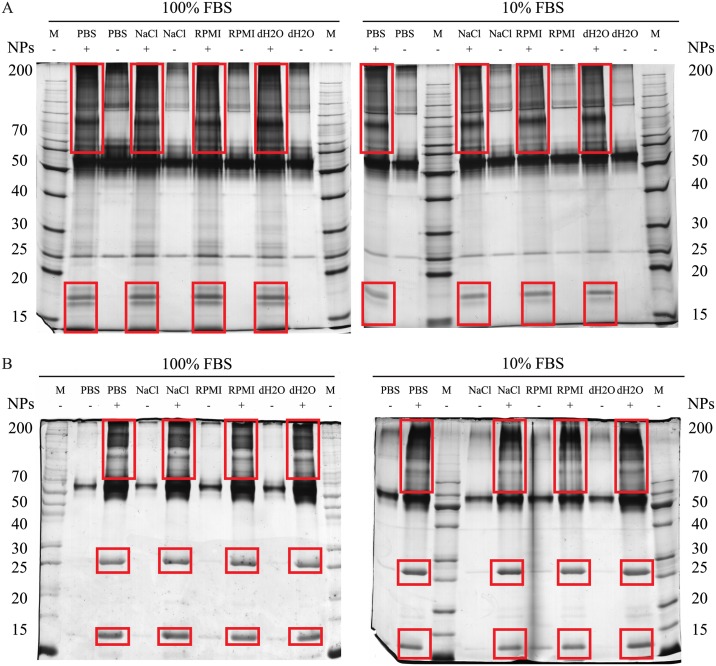
Analysis of NPs protein corona using SDS-PAGE. PAA (A) and silica (B) NPs were dispersed in different media (dH_2_O, NaCl, PBS, RPMI) and incubated for 1 h in 10% or 100% FBS. Samples denoted with (+) contained PAA (A) or silica (B) NPs. In samples without NPs (-) were the proteins which adhered to plastic despite thorough washing. Only the bands that were different in respective test and control sample (mobbed red) were excised and analysed by MS. M denotes lanes loaded with molecular mass standards. Molecular mass is given in kDa.

As described in the Methods section, NPs were dispersed in four different media: PBS, NaCl, RPMI and dH_2_O. Properties of the media are given in Table 1. Protein corona was formed by exposure of NPs to FBS at two different concentrations: 10% and 100% stock solution. After the separation of NPs from FBS, proteins were separated from NPs by exposure to SDS and heat (95°C, 5 min), followed by subsequent centrifugation. Afterwards, proteins were separated on 10% SDS-PAGE gel under non-reducing conditions (Fig 2). Despite the three washing steps, some proteins were still detected in control samples, where NPs were not present (lanes denoted by subtraction symbol), due to adhesion to plasticware. Nonetheless, SDS-PAGE showed the presence of proteins interacting exclusively with PAA or silica NPs, compared to controls. However, results as presented in Fig 2 did not reveal any visible differences in the protein corona composition between the same NPs dispersed in different media. Also, the concentration of FBS used to prepare the corona seemed to have no obvious effect on corona composition. In order to confirm results from SDS-PAGE, we identified the proteins of each sample separately by MS. To avoid misleading results, we analysed only the protein bands that clearly differed between the samples or were not present in the controls.

### Characterisation of NPs protein corona

**Fig 3:**
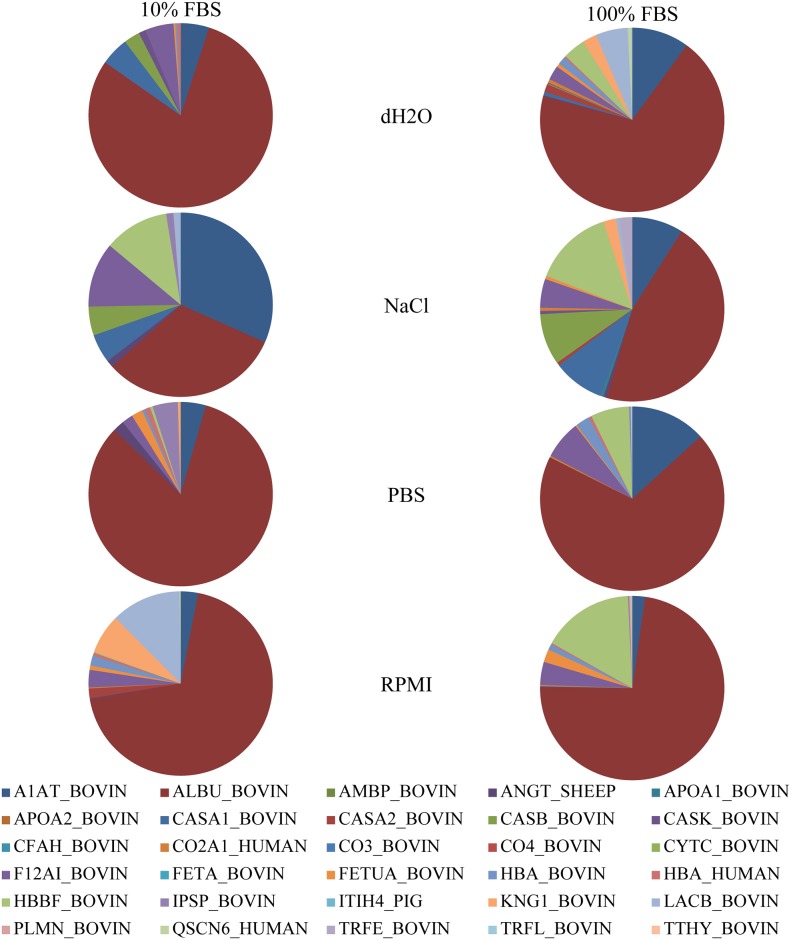
Relative abundance of proteins identified in samples of PAA NPs dispersed in different media. PAA NPs were dispersed in different media and incubated for 1h in 10% or 100% FBS. Proteins were separated from NPs, analysed on SDS-PAGE and identified by MS. Spectral counts were used as a measure of individual protein in a sample. Legend is further explained in Table 3. The differences in protein corona composition of PAA NPs dispersed in PBS, NaCl and RPMI-1640 were significant compared to protein corona of PAA NPs dispersed in dH2O (p < 0.05).

**Fig 4:**
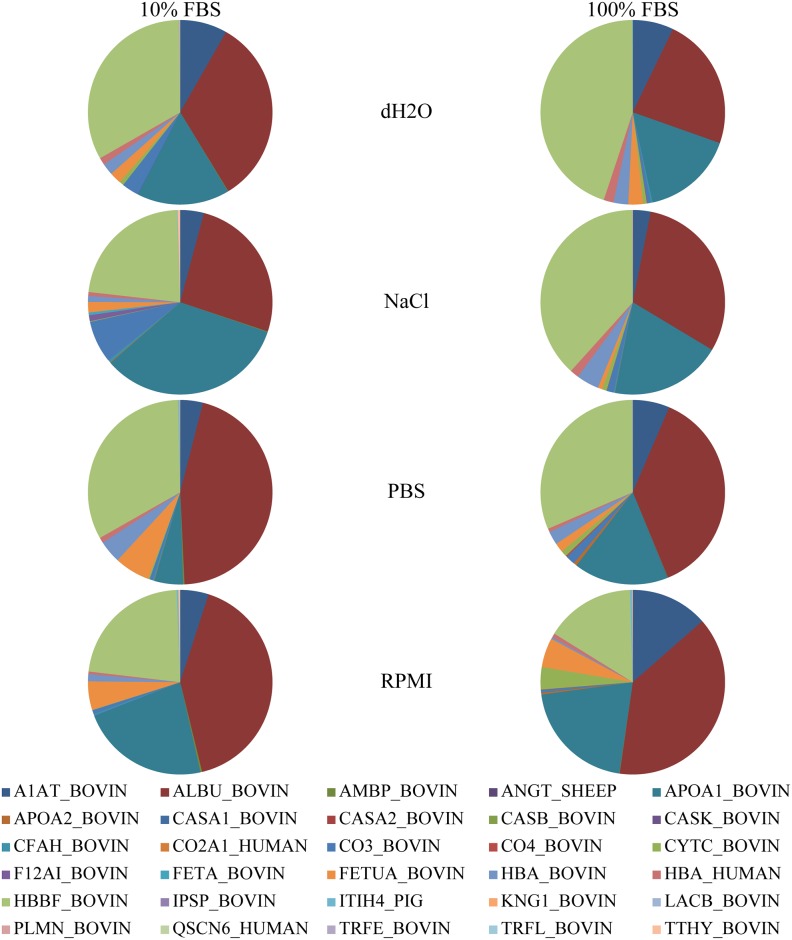
Relative abundance of proteins identified in samples of silica NPs dispersed in different media. Silica NPs were dispersed in different media and incubated for 1h in 10% or 100% FBS. Proteins were separated from NPs, analysed on SDS-PAGE and identified by MS. Spectral counts were used as a measure of individual protein in a sample. Legend is further explained in Table 3. The differences in protein corona composition of silica NPs dispersed in PBS, NaCl and RPMI-1640 were significant compared to protein corona of silica NPs dispersed in dH2O (p < 0.05).

Composition of NPs protein corona was analysed by MS. A complete list of identified proteins, their calculated pIs, molecular masses and biological functions are listed in Table 3. Relative amounts of corona proteins in different NP formulations are shown in Figs 3 and 4. Differences in relative protein representation in coronas of PAA and silica NPs dispersed in different dispersion media (dH_2_O, NaCl, PBS, RPMI) and incubated in different concentrations of FBS (10 and 100%) are obvious. Serum albumin (ALBU_BOVIN) and α-1-antiproteinase (A1AT_BOVIN) were the only two proteins found in all samples. The hemoglobin fetal subunit β (HBBF_BOVIN) was found in all samples but in the sample with PAA NPs prepared in dH_2_O and incubated in 10% FBS, while α-2-HS-glycoprotein (FETUA_BOVIN) was absent only in the sample with PAA NPs prepared in NaCl and incubated in 10% FBS. Independent of the media and FBS concentration during the preparation of the NPs samples, α-S2-casein, β-casein, κ-casein, collagen α-1(II) chain, plasma serine protease inhibitor, kininogen-1, β-lactoglobulin, sulfhydryl oxidase 1, serotransferrin and lactotransferrin were found only in samples prepared with PAA NPs. Proteins characteristically associated only with the silica NPs coronas, independent of the media and FBS concentration for their preparation, were AMBP, apolipoprotein A-II, complement factor H, complement C3 and C4, cystatin C, α-fetoprotein, inter-α-trypsin inhibitor heavy chain H4, plasminogen and transthyretin. Interestingly, complement C4 (CO4_BOVIN) was present exclusively in the corona of silica NPs prepared in 100% FBS, no matter which media was used in the process. There were no proteins exclusive for the particular type of dispersion media used to prepare NPs. However, there were substantial differences in relative amount of individual proteins found in corona of NPs dispersed in different media.

**Table 3:** List of serum proteins found in the corona of PAA and silica NPs.

Protein accession	Theoretical pI	Molecular mass (Da)	Protein name	Biological process	Molecular function
**A1AT_BOVIN**	5.98	43693.91	α1-antiproteinase	Protein inhibition	Endopeptidase inhibitor
**ALBU_BOVIN**	5.60	66432.96	Serum albumin	Transport	Binds water, cations, fatty acids, hormones, bilirubin and drugs
**AMBP_BOVIN**	5.26	16197.25	Protein AMBP†	Protein inhibition	Serine protease inhibitor
**ANGT_SHEEP**	6.56	49080.32	Angiotensinogen	Hemostasis	Regulator of blood pressure
**APOA1_BOVIN**	5.36	27549.08	Apolipoprotein A-I	Transport	Cholesterol transport
**APOA2_BOVIN**	5.34	8722.76	Apolipoprotein A-II†	Transport	Lipid binding; antimicrobial activity
**CASA1_BOVIN**	4.91	22974.87	α-S1-casein	Transport	Calcium phosphate transport
**CASA2_BOVIN**	8.34	24348.55	α-S2-casein*	Transport	Calcium phosphate transport
**CASB_BOVIN**	5.13	23583.29	β-casein*	Transport	Calcium ion transport
**CASK_BOVIN**	5.93	18974.42	κ -casein*	Tissue structuring	Identical protein binding
**CFAH_BOVIN**	6.33	138259.19	Complement factor H†	Immune system	Complement activation (alternative pathway)
**CO2A1_HUMAN**	9.14	96052.84	Collagen α-1(II) chain*	Tissue structuring	Extracellular matrix structural constituent
**CO3_BOVIN**	6.37	185047.41	Complement C3†	Immune system	Complement system activation
**CO4_BOVIN**	5.55	67995.42	Complement C4†	Immune system	Propagation of the classical complement pathway
**CYTC_BOVIN**	9.03	13412.30	Cystatin-C†	Protein inhibition	Endopeptidase inhibitor activity
**F12AI_BOVIN**	6.08	49273.26	Factor XIIa inhibitor	Hemostasis	Serine protease inhibitor
**FETA_BOVIN**	5.92	66412.03	α-fetoprotein†	Transport	Binds copper, nickel, and fatty acids
**FETUA_BOVIN**	5.10	36353.24	α-2-HS-glycoprotein	Immune system	Cysteine-type endopeptidase inhibitor activity
**HBA_BOVIN**	8.19	15053.18	Hemoglobin subunit α	Transport	Oxygen transporter activity
**HBA_HUMAN**	8.73	15126.36	Hemoglobin subunit α	Transport	Oxygen transporter activity
**HBBF_BOVIN**	6.51	15859.23	Hemoglobin fetal subunit β	Transport	Oxygen transporter activity
**IPSP_BOVIN**	9.12	42495.23	Plasma serine protease inhibitor*	Transport	Heparin binding; retinoic acid binding
**ITIH4_PIG**	6.35	99349.79	Inter-α-trypsin inhibitor heavy chain H4†	Immune system	Protease inhibitor
**KNG1_BOVIN**	6.05	66845.54	Kininogen-1*	Hemostasis	Endopeptidase inhibitor activity
**LACB_BOVIN**	4.83	18281.21	β-lactoglobulin*	Transport	Retinol binding
**PLMN_BOVIN**	7.39	88393.49	Plasminogen†	Hemostasis	Dissolves fibrin
**QSCN6_HUMAN**	9.05	79578.10	Sulfhydryl oxidase 1*	Hemostasis	Oxidation of sulfhydryl groups in peptide and protein thiols
**TRFE_BOVIN**	6.50	75829.68	Serotransferrin*	Transport	Ferric iron binding
**TRFL_BOVIN**	8.67	76143.90	Lactotransferrin*	Transport	Ferric ion binding
**TTHY_BOVIN**	5.91	13557.31	Transthyretin†	Transport	Thyroid hormone binding

Protein accessions are alphabetically ordered. Proteins present exclusively in the corona of PAA NPs are designated by * while those present exclusively in the corona of silica NPs by †.

In the corona of PAA NPs, the largest differences in relative protein abundance were found in the case of serum albumin (ALBU_BOVIN) and α-1-antiproteinase (A1AT_BOVIN) which were also the most abundant proteins in the corona of PAA NPs. The third most abundant protein in PAA NP coronas was the hemoglobin fetal subunit β (HBBF_BOVIN) while factor XIIa inhibitor (F12AI_BOVIN) was the fourth most abundant protein. The medium that mostly affected the changes in relative abundances of these proteins in the corona of PAA NPs was NaCl. Markedly, the addition of NaCl to dispersion media also increased the ratio of α-S1-casein (CASA1_BOVIN) and β-casein (CASB_BOVIN) in PAA NP coronas.

Furthermore, quantitative protein ratios in coronas of silica NPs were not the same as in coronas of PAA NPs. Serum albumin (ALBU_BOVIN) was on average the most abundant protein also in coronas of silica NPs while the second most abundant protein in these kind of NPs was hemoglobin fetal subunit β (HBBF_BOVIN). Apolipoprotein A-I (APOA1_BOVIN) was the third most abundant protein on average, but, interestingly, also the second most variable protein in coronas of alternatively prepared silica NPs. The protein that also importantly contributed to silica NPs protein corona composition was α-1-antiproteinase (A1AT_BOVIN). The influence of the type of dispersion media on the corona composition is obviously more complex in the case of silica NPs than at PAA NPs.

### Properties of identified proteins

**Fig 5:**
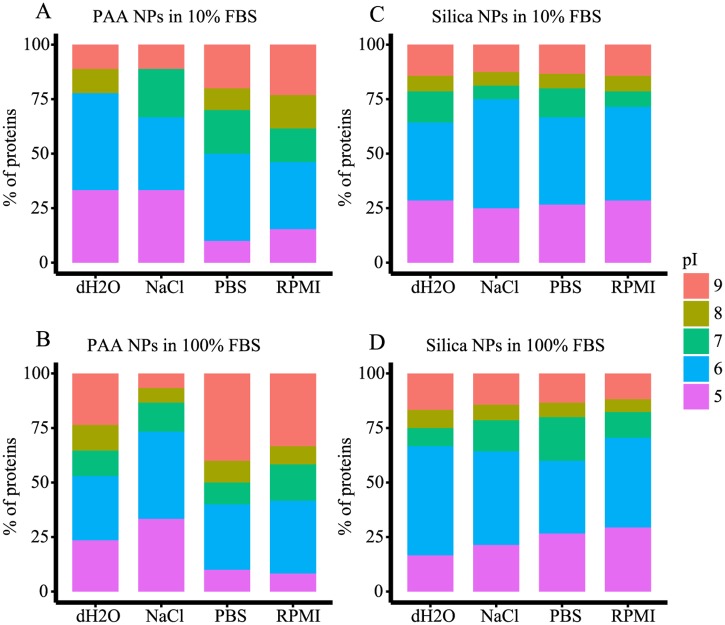
NPs protein coronas by theoretical pI of their constituting proteins. PAA (A, B) and silica (C, D) NPs were dispersed in different media and incubated for 1 h in 10% (A, C) or 100% (B, D) FBS. Proteins constituting NPs coronas were separated by SDS-PAGE and identified by MS. Theoretical pIs were calculated using Compute pI/Mw tool from the ExPASy portal.

To further understand the differences in protein corona composition of PAA and silica NPs dispersed in different media, we compared theoretical pIs and GO descriptions of proteins in protein corona (Table 3). In Fig 5 we show the distribution of proteins, constituents of alternatively prepared NP coronas, according to their theoretical pI. Majority of proteins (55–80%) found in coronas of PAA and silica NPs were negatively charged at the physiological pH. Indicatively, more positively charged proteins were found in coronas of PAA NPs (Fig 5A and 5B) than of silica NPs (Fig 5C and 5D). In the case of PAA NPs, type of dispersion media and serum concentration evidently directed the composition of protein corona (Fig 5A and 5B). Theoretical pIs of proteins detected in the coronas of silica NPs are much less dependent on conditions under which coronas were formed (Fig 5C and 5D).

**Fig 6:**
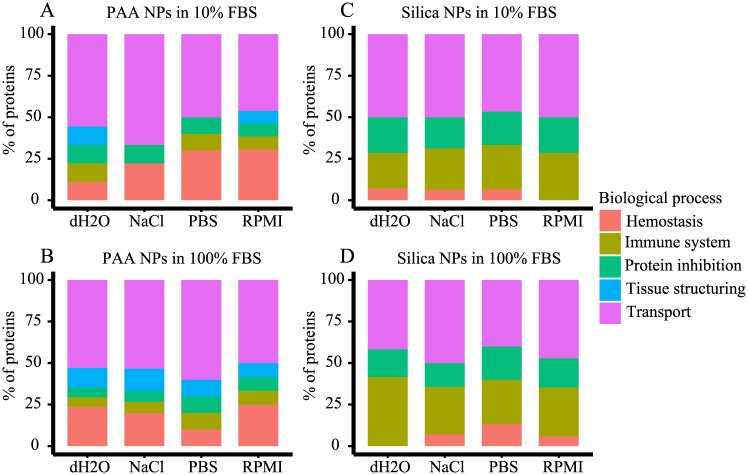
NPs protein coronas by Gene Ontology (GO) biological process of constituting proteins. PAA (A, B) and silica (C, D) NPs were dispersed in different media and incubated for 1h in 10% (A, C) or 100% (B, D) FBS. Proteins forming corona of NPs were separated by SDS-PAGE and characterized by MS. GO information was acquired from the UniProt database.

Molecular function of individual proteins and biological processes where these proteins are involved were obtained from UniProt database (Table 3). Proteins were grouped according to five major GO biological processes: hemostasis, immune system, protein inhibition, tissue structuring and transport. In Fig 6 we display the distribution of proteins, constituents of alternatively prepared NP coronas, according to participation in the above-mentioned biological processes. Transport was the process in which the majority of proteins from either PAA or silica NP coronas were involved, no matter of the type of the dispersion media and FBS concentration used for the preparation of samples. The second most frequent biological process in which the identified corona proteins were involved is hemostasis in the case of PAA NPs (Fig 6A and 6B) and the immune response in the case of silica NPs (Fig 6C and 6D). Interestingly, none of silica NPs corona protein belongs among tissue structuring proteins. Such proteins were however present in most coronas of PAA NPs. The GO profiles of proteins detected in coronas of PAA NPs were much more dependent on the FBS concentration and the type of dispersion media used for corona formation (Fig 6) than in the case of silica NPs.

## Discussion

In the presented study we tested and confirmed the hypothesis that dispersion media considerably contributes to protein composition of NPs corona. We used two types of NPs: i) magnetic cobalt-ferrite poly-acrylic acid coated NPs (PAA NPs) as a representative biomedical NPs, potentially useful for imaging or hyperthermia, and ii) commercially available silica NPs that are produced on a large scale and are suitable for industrial use (e.g. pigment production, electronics industry). Silica is also often used as a coating of NPs for biomedical applications [44,45]. While biomedical NPs are designed for medical use and come in contact with the human tissues directly, industrial NPs interact with the human organism only unintentionally.

Prior to use, NPs are usually dispersed in some specific type of media [46]. Media chosen for dispersion of NPs in this study are routinely used in almost all cell culture laboratories and could potentially be used as dispersion media for NPs used in in vitro experiments. Due to relevance for in vitro experiments, protein corona was formed by the incubation of NPs in 10% FBS since the same concentration of FBS is often used in cell culturing. Because it was shown that FBS concentration may affect protein corona composition [47], we also used 100% FBS for comparison. Higher concentration of FBS is also more relevant for physiological conditions.

By physico-chemical and proteomic characterization we demonstrated that the type of the dispersion media used to prepare NPs was relevant for protein corona composition. The media used for dispersion of PAA NPs had little effect on size of these NPs (Table 2) while counter ions from complex media like PBS and RPMI significantly decreased absolute value of the zeta potential from -59 mV to an average of 28 mV due to screening effects. On the other hand, dispersion media had strong effect on the measurable size values of hydrodynamic units formed from silica NPs, especially through the effect of agglomeration. Measured low values of absolute zeta potentials confirmed such conclusions.

When NPs come in contact with proteins, some of the proteins adhere to NPs surface [9,10]. To demonstrate the effect of proteins on the hydrodynamic diameter and zeta potential of NPs, we performed the characterization of NPs also after the protein corona formation in 10% FBS. As expected, protein corona formation in 10% FBS increased hydrodynamic diameter of PAA NPs dispersed in all four media used. More specifically Z-average size was increased for all four media while when hydrodynamic diameter based on particle number distribution is analysed, we have obtained decreased values (Table 2). It is important to note that Z-average size includes information on entire population of measured NPs (including agglomerates), while number-based hydrodynamic diameter lacks information about small number of agglomerates that were formed in the presence of FBS. It must be emphasized, that the differences between samples prepared in different media, compared to samples prepared in dH2O were statistically insignificant and we can only conclude that effect of protein corona on hydrodynamic size of PAA NPs was small.

Regardless of the media used for dispersion, zeta potential of PAA NPs converged to an average value of -16 ± 3 mV when proteins were present, which was comparable to our previous results [48]. Reduced absolute zeta potential was the result of protein corona which shielded carboxyl groups on the surface of PAA NPs. The final, very similar value of zeta potential thus demonstrates that zeta potential in media with 10% FBS was dominated by the presence of strongly charged proteins present in the outer shell due to electrostatic interactions, while in media without FBS the ion composition (i.e. ionic strength, type and concentration) determined the zeta potential.

From the results of silica NPs characterization in different media, we can conclude that agglomeration of silica NPs was present. Silica NPs used in this study were prepared for industrial use, which seldom requires stability of dispersed NPs in complex media. Z-average size and zeta potential results were clearly affected by agglomeration, despite our effort for applying identical kinetic conditions for each measurement. Consequently, the agglomeration of silica NPs was even more evident when size and zeta potential of silica NPs were measured in media with 10% FBS. We also performed measurements in 100% FBS, but clearly the signal of proteins, mostly albumin, dominated all other signals, thus such measurement do not resemble characteristics of NPs. This effect was already observed for measurements of silica NP-characteristics in 10% FBS (Table 2).

To confirm that proteins adhered to NPs surface and to further analyse them we performed SDS-PAGE analysis. NPs with the adhered material were separated from the dispersion solution by centrifugation. The NPs-bound material was desorbed into the SDS-containing buffer and was analysed with SDS-PAGE [40]. Results shown in Fig 2 confirmed the presence of proteins in coronas on all tested NPs samples. Surprisingly, despite thorough washing, highly sensitive silver staining revealed traces of proteins also in control samples to which NPs were not added. This can be explained by non-specific adherence of proteins to plastic [49]. Numerous washings of microcentrifuge tubes could reduce this background signal, but this would also change the profile of protein coronas in samples with NPs. Nonetheless, specific protein bands that were present only in samples with NPs confirmed that protein corona was formed during the incubation of NPs with FBS. Visual comparison of intensities of protein bands on the gels revealed differences between PAA NPs and silica NPs. Because we observed differences in band intensities, we also determined total protein content in our samples. Control samples contained 319.5 ± 99.5 μg of protein per ml (S4 Fig). Differences in the total protein content between silica and PAA NPs prepared in different media were insignificant. In general, we can say that PAA NPs bound relatively small amounts of proteins (S4A Fig), while silica NPs displayed higher protein-binding capacity (S4B Fig). In accordance with the change in hydrodynamic diameter (Table 2), the amount of the NPs-attached proteins was the largest when NPs were dispersed in dH_2_O (S4 Fig). We can explain such effect by the absence of counter ion layer in water, which reduced electrostatic interactions between NPs and proteins in other media.

To analyse the NPs protein corona composition even further, we identified individual proteins in the corona of PAA and silica NPs dispersed in different media by MS [25]. Relative protein abundances calculated from the MS data confirmed that the composition of the corona clearly depended on the type of NP (Figs 3 and 4). Ten proteins were found exclusively in the corona of PAA NPs and ten proteins exclusively in the corona of silica NPs (Table 3). Ten proteins were common to coronas of both NPs, out of which four, i.e. serum albumin, α-1-antiproteinase, α-2-HS-glycoprotein and hemoglobin fetal subunit β, were detected also in control samples (S5 Fig). Fraction of the individual proteins found in the corona of PAA and silica NPs was not equal to the fraction of these proteins in FBS [50]. The concentration of FBS to which the NPs were exposed also governed the NPs protein corona composition to some extent. Proteins that were detected in coronas only when the NPs were incubated in 100% FBS were α-S2-casein, collagen α-1(II) chain, serotransferrrin and lactotransferrin for PAA NPs and complement C4 for silica NPs. Due to relatively low abundance of those proteins in coronas, we assume that after the exposure of NPs to 10% FBS these proteins simply adhered to NPs in quantity below the detection limit of our method.

Importantly, protein composition of the corona was the function of the media used for dispersion of the NPs in addition to the known dependence on the type of NPs and the FBS concentration. Furthermore, it is interesting to note the differences observed in protein corona composition when comparing more complex PBS and RPMI media versus NaCl and dH2O media (Figs 3 and 4). It is important to mention that there is a primary corona formed in the complex media like RPMI composed of macromolecules, small molecules and counter ions [51]. This consequently leads to at least two effects: i) unspecific effect through the change of zeta potential (decreased zeta potential due to larger screening effect in RPMI and PBS due to counter ions) which consequently can influence which proteins will more preferentially bind when exposed to proteins in serum and ii) the other more specific where there is a possibility that some specific molecules from so called primary corona of macromolecules can modulate binding of some specific proteins. However, since the zeta potential was very similar for PAA NPs dispersed in NaCl, PBS, and RPMI and significantly different for PAA NPs dispersed in dH2O, while the protein composition patterns (Figs 3 and 4) shows similarity between dH2O and NaCl and similarity between RPMI and PBS, clearly the change of zeta potential is not the dominant effect for corona composition. Furthermore, the change in zeta potential measured in in different media with respect to the pure water correlated with the increasing ionic strength of the media (Table 1).

Differences in the protein corona composition can be responsible for different effects of NPs observed in vitro, for example the cellular uptake of NPs [14,30,34]. It is also known from the previous studies that already a single protein type adhered to NPs surface can trigger biologically-relevant effect such as inflammation [21,23]. Our results revealed that dispersion media used to prepare NPs determined the protein structure of the corona in qualitative and quantitative way. In PAA NPs samples, for example, casein proteins were present in coronas in high quantities only when NPs were dispersed in dH_2_O or NaCl, but not when NPs were dispersed in more complex dispersion media, PBS or RPMI, which contained divalent ions and even macromolecules (RPMI). On the other hand, dispersion of silica NPs in different media did not substantially changed the profile of proteins bound to their surface, only the ratios between adhered proteins in individual samples were altered. This effect can be described as a function of dispersion media composition, i.e. RPMI contains macromolecules which change the surface of NPs trough electrostatic interactions between NPs and macromolecule surface groups, but the actual mechanisms are not trivial and further research is needed to explain them in detail.

Proteins found in the corona of NPs displayed an array of functions and are implicated in different biological processes. Regardless of dispersion media, FBS concentration and the type of NPs, the proteins identified in coronas are mainly involved in transport (Table 3 and Fig 6). Interestingly, there was an important difference between both types of NPs regarding the presence of immune system-related proteins in their coronas. While PAA NPs bound only one immune system-related protein, α-2-HS-glycoprotein, corona of silica NPs contained also three others, complement factor H, complement C3 and complement C4. The latter are all part of the complex complement system, an important component of the innate immunity [52]. Relative abundance of complement C3 protein in corona was increased when silica NPs were dispersed in NaCl, which further implies the relevance of the dispersion media, used to prepare NPs, for the characteristics of the NPs preparation. There was almost complete absence of apolipoproteins in the corona of PAA NPs, with the exception of apolipoprotein A-I found in the corona of NPs dispersed in NaCl. Although there are some implications that apolipoprotein A-I is connected to bioaccumulation of NPs in cells [7] our PAA NPs were shown to accumulate in different cell lines despite of the absence of this specific protein in their corona [38,53]. Again, dispersion media proved to be a relevant factor in determining the NPs protein corona composition.

## Conclusions

In this study we analysed different factors that influenced the composition of protein corona of NPs. We demonstrated that type of the dispersion media in addition to the selected NPs type very importantly determines binding of proteins to NPs surface. The type of dispersion media also importantly dictated the relative abundancies of individual proteins in NPs corona: patterns were similar for NPs dispersed in dH2O and NaCl or for NPs dispersed in PBS and RPMI. It is important to note that the protein corona of silica NPs contained three complement system-related proteins: complement factor H, complement C3 and complement C4. Although abundancies of those proteins were sometimes relatively small, they could play an important role in the context of, for example, immune response. We believe that dispersion media is an important factor to consider in further studies of the protein corona and may also be acknowledged in retrospective for the studies already performed.

## Supporting Information

S1 Fig TEM micrograph of PAA NPs dispersed in water. Scale bar: 100 nm.
(TIF)

S2 Fig Analysis of 10% FBS with SDS-PAGE. M denotes the lane loaded with the protein mass standards (molecular masses are in kDa).
(TIF)

S3 Fig Relative abundance of proteins identified in individual NP samples. PAA and silica NPs were dispersed in different media and incubated for 1h in 10% or 100% FBS. Proteins were separated from NPs, analysed on SDS-PAGE and identified by MS. Spectral counts were used as a measure of individual protein in a sample. White space indicates the absence of a protein in a sample. Protein accessions are ordered alphabetically. Accessions are further explained in Table 1. NPs formulations are coded as: type of NPs—dispersion media—% of FBS (e.g. PAA—NaCl—100 designates PAA NPs prepared in NaCl and incubated in 100% FBS).
(TIF)

S4 Fig Quantitation of total proteins in individual samples. PAA (A) and silica (B) NPs were dispersed in different media and incubated for 1h in 10% or 100% FBS. Proteins were separated from NPs and total protein quantity was measured using Pierce™ 660 nm assay. Data points are shown in black. Mean values with standard error of the mean from at least three independent replicates are shown in red. Note that media used for dispersion did not affect total protein quantity of control samples.
(TIF)

S5 Fig Percentage of total proteins identified in individual control samples. Dispersion media without NPs were incubated for 1h in 10% or 100% FBS. Proteins that stayed adhered to microcentrifuge tube after three washing steps were analysed on SDS-PAGE and identified with MS. Spectral counts were used as a measure of individual protein in a sample. White space denotes absence of a protein in a sample. Please note: short names of proteins are explained in S1 Table. Samples are coded as: dispersion media—% of FBS (*e*.*g*. sample NaCl—100 was prepared with sodium chloride and incubated in 100% FBS).
(TIF)

S6 Fig Dynamic light scattering results for PAA NPs dispersed in distilled water. Distributions based on intensity (A), number (B), and volume (C) are shown. This result is based on twenty consecutive measurements of one sample.
(TIF)

S7 Fig Dynamic light scattering results for silica NPs dispersed in ethanol. Distributions based on intensity (A), number (B), and volume (C), are shown. This result is based on twenty consecutive measurements of one sample.
(TIF)

S1 Table List of serum proteins found in the control samples. Please note: protein accessions are alphabetically ordered.
(PDF)

S1 Text Composition of RPMI-1640 media. (PDF)
