# Supplementary figures and images for: Dispersion of Nanoparticles in Different Media Importantly Determines the Composition of Their Protein Corona
Source: PLoS One. 2017 Jan 4;12(1):e0169552. doi: 10.1371/journal.pone.0169552 (PMC5215476; doi:10.1371/journal.pone.0169552)

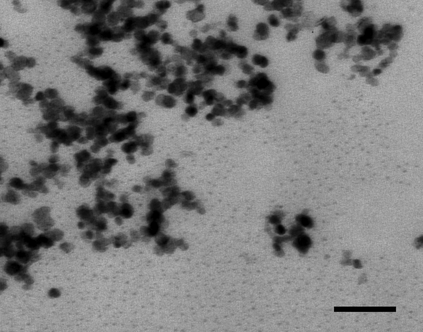

Supplement: S1 Fig — Scale bar: 100 nm. (TIF) [file pone.0169552.s001.tif]

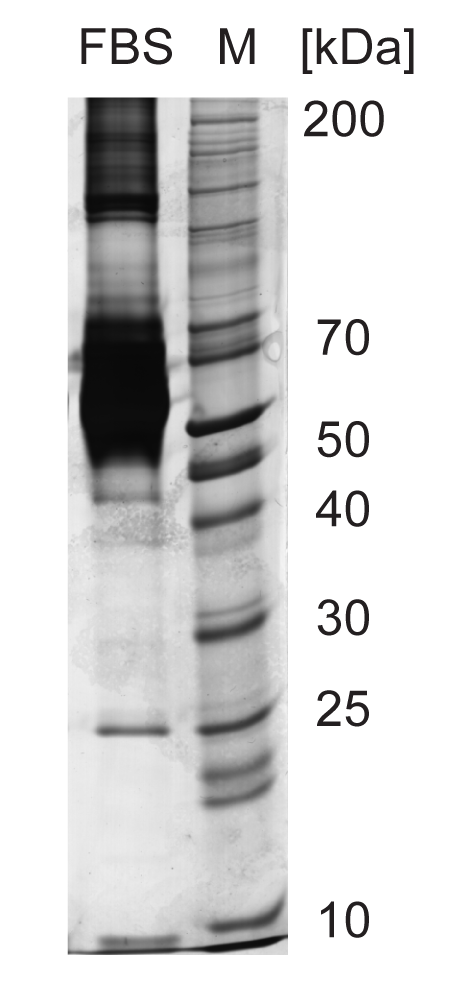

Supplement: S2 Fig — M denotes the lane loaded with the protein mass standards (molecular masses are in kDa). (TIF) [file pone.0169552.s002.tif]

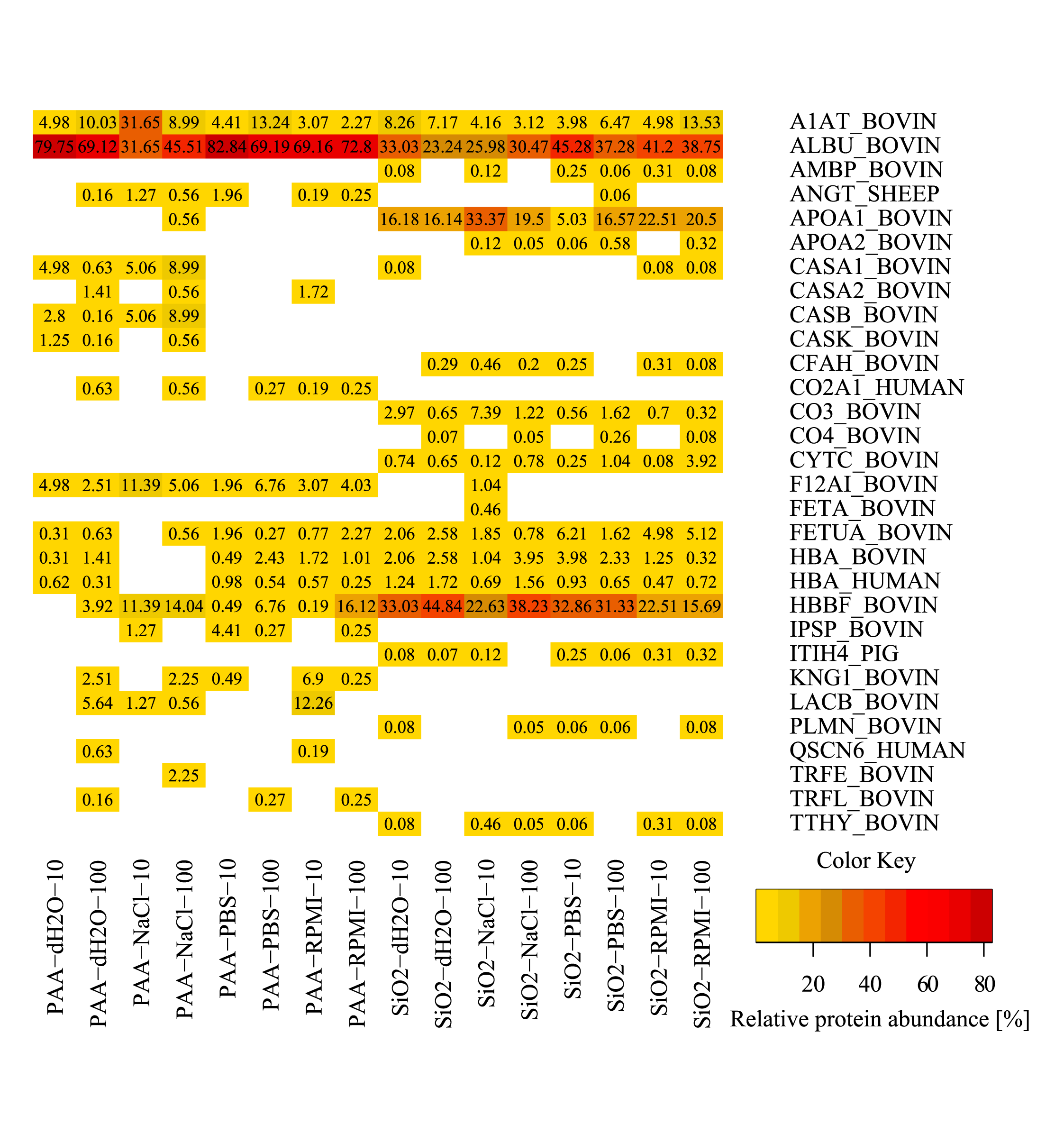

Supplement: S3 Fig — PAA and silica NPs were dispersed in different media and incubated for 1h in 10% or 100% FBS. Proteins were separated from NPs, analysed on SDS-PAGE and identified by MS. Spectral counts were used as a measure of individual protein in a sample. White space indicates the absence of a protein in a sample. Protein accessions are ordered alphabetically. Accessions are further explained in Table 1. NPs formulations are coded as: type of NPs—dispersion media—% of FBS (e.g. PAA—NaCl—100 designates PAA NPs prepared in NaCl and incubated in 100% FBS). (TIF) [file pone.0169552.s003.tif]

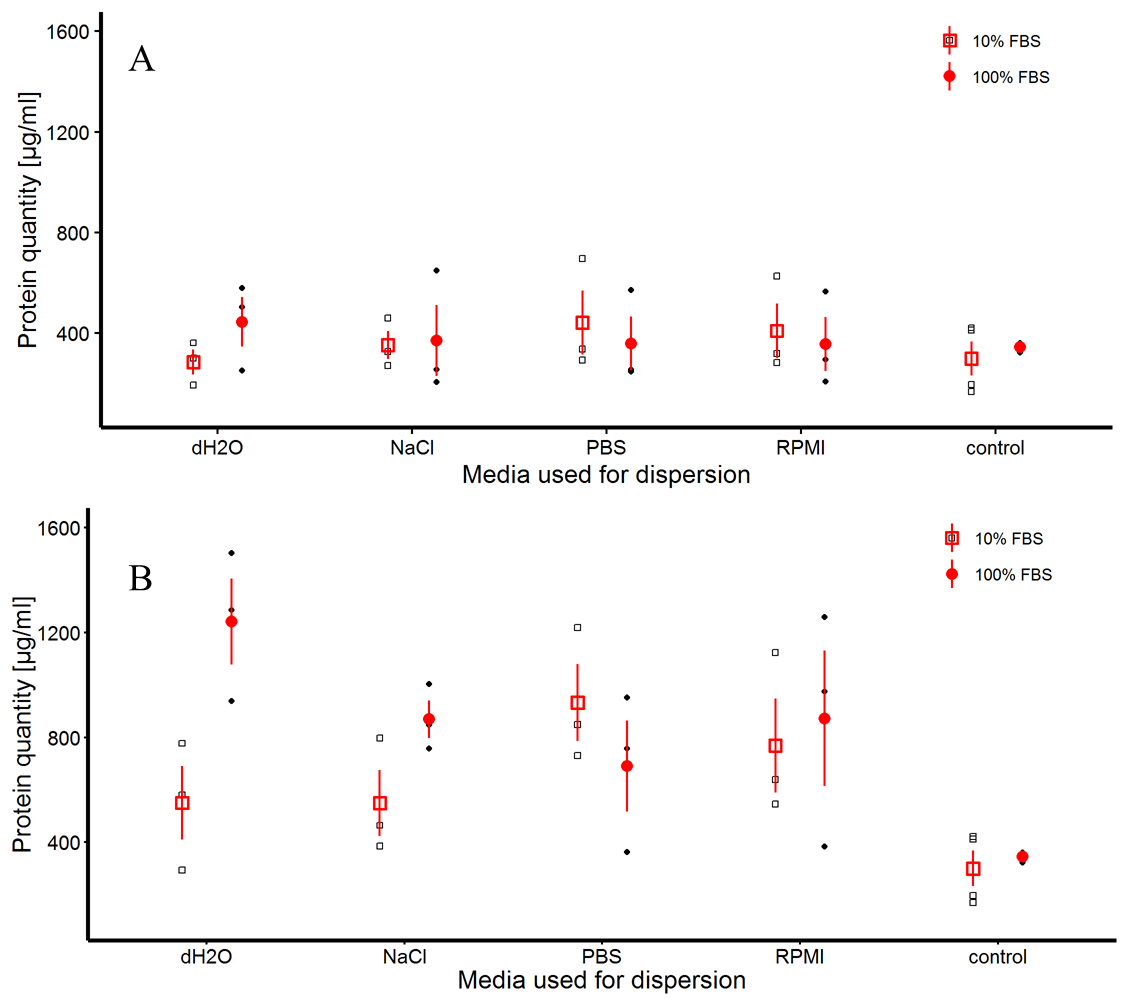

Supplement: S4 Fig — PAA (A) and silica (B) NPs were dispersed in different media and incubated for 1h in 10% or 100% FBS. Proteins were separated from NPs and total protein quantity was measured using Pierce™ 660 nm assay. Data points are shown in black. Mean values with standard error of the mean from at least three independent replicates are shown in red. Note that media used for dispersion did not affect total protein quantity of control samples. (TIF) [file pone.0169552.s004.tif]

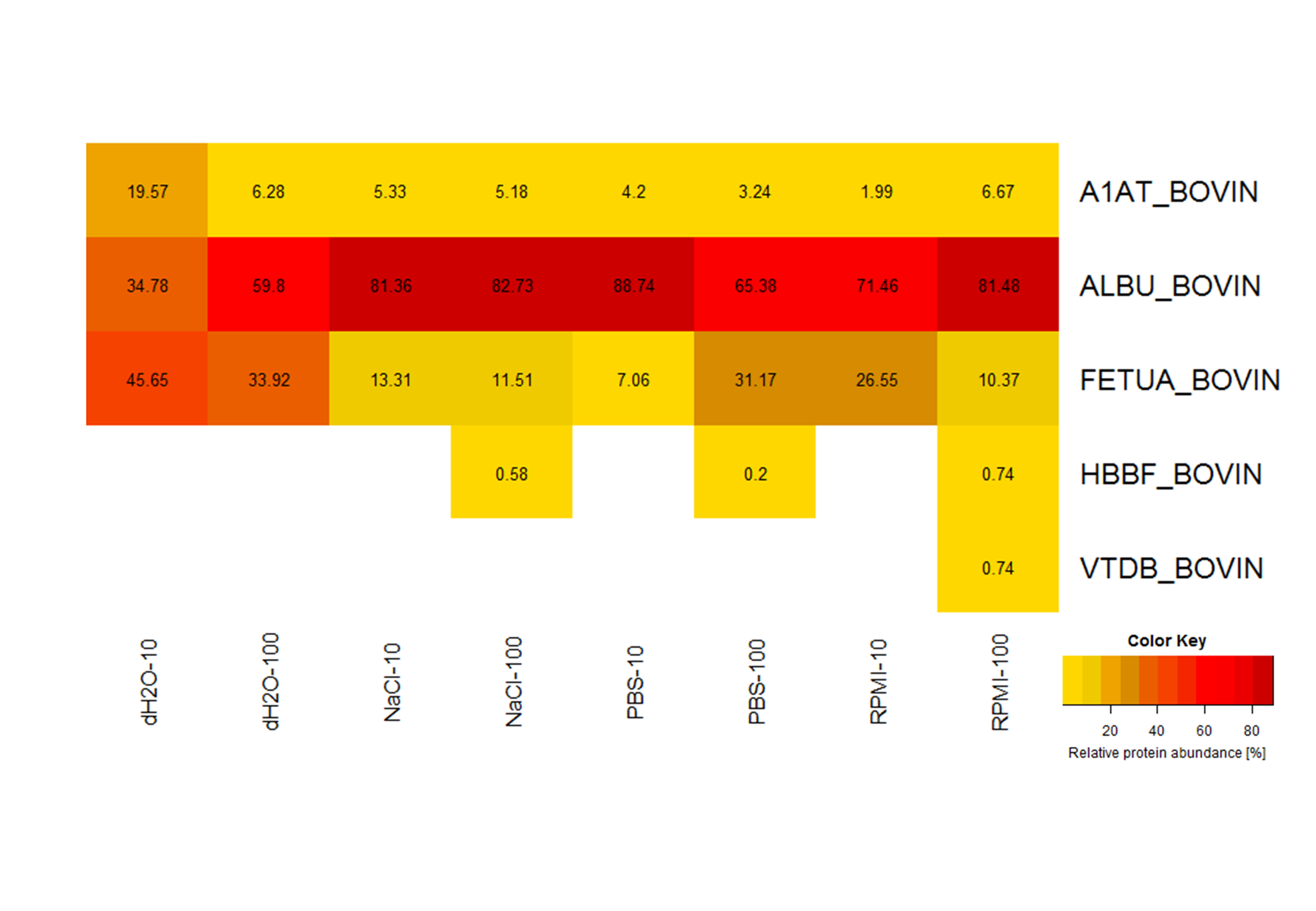

Supplement: S5 Fig — Dispersion media without NPs were incubated for 1h in 10% or 100% FBS. Proteins that stayed adhered to microcentrifuge tube after three washing steps were analysed on SDS-PAGE and identified with MS. Spectral counts were used as a measure of individual protein in a sample. White space denotes absence of a protein in a sample. Please note: short names of proteins are explained in S1 Table. Samples are coded as: dispersion media—% of FBS (e.g. sample NaCl—100 was prepared with sodium chloride and incubated in 100% FBS). (TIF) [file pone.0169552.s005.tif]

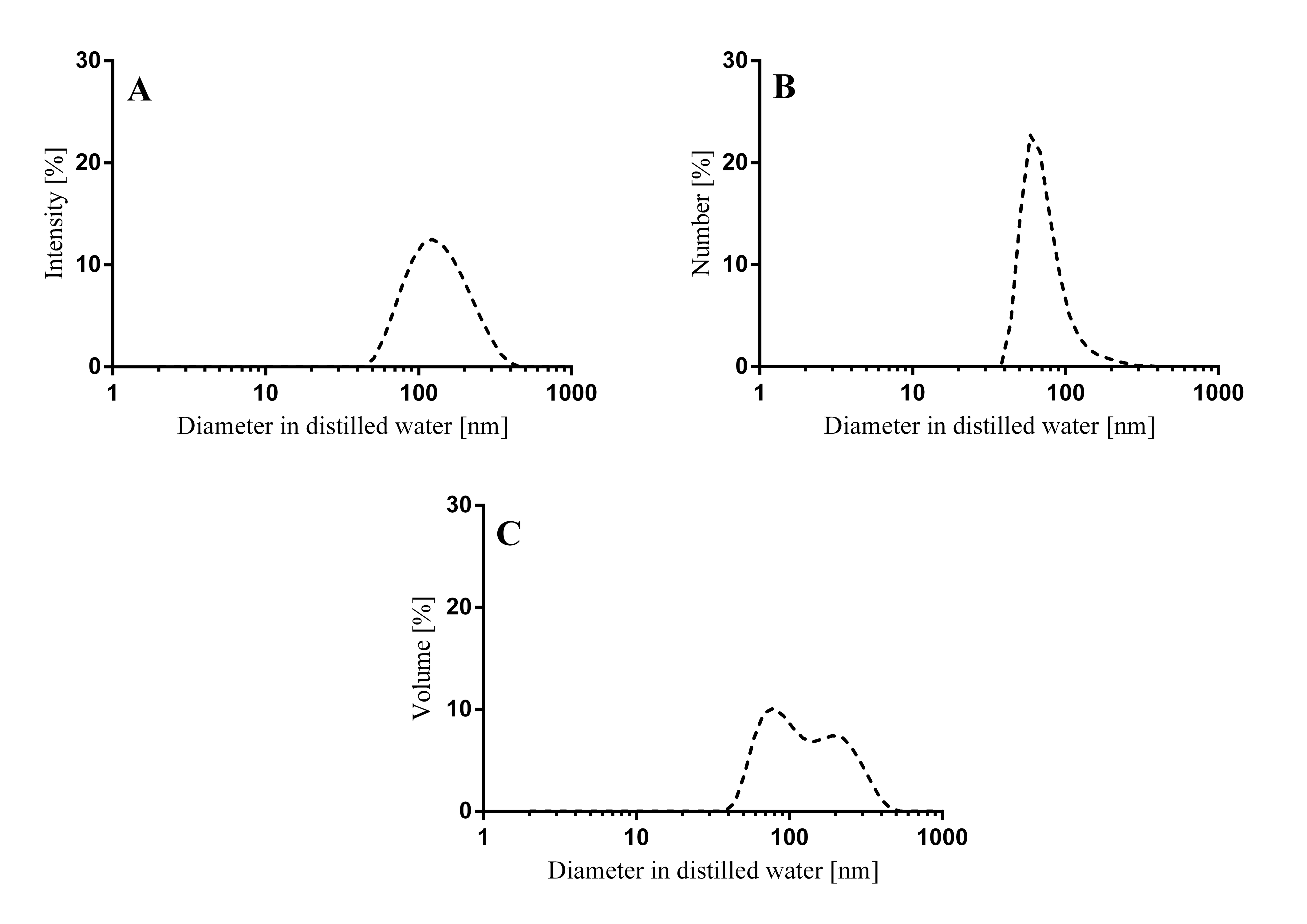

Supplement: S6 Fig — Distributions based on intensity (A), number (B), and volume (C) are shown. This result is based on twenty consecutive measurements of one sample. (TIF) [file pone.0169552.s006.tif]

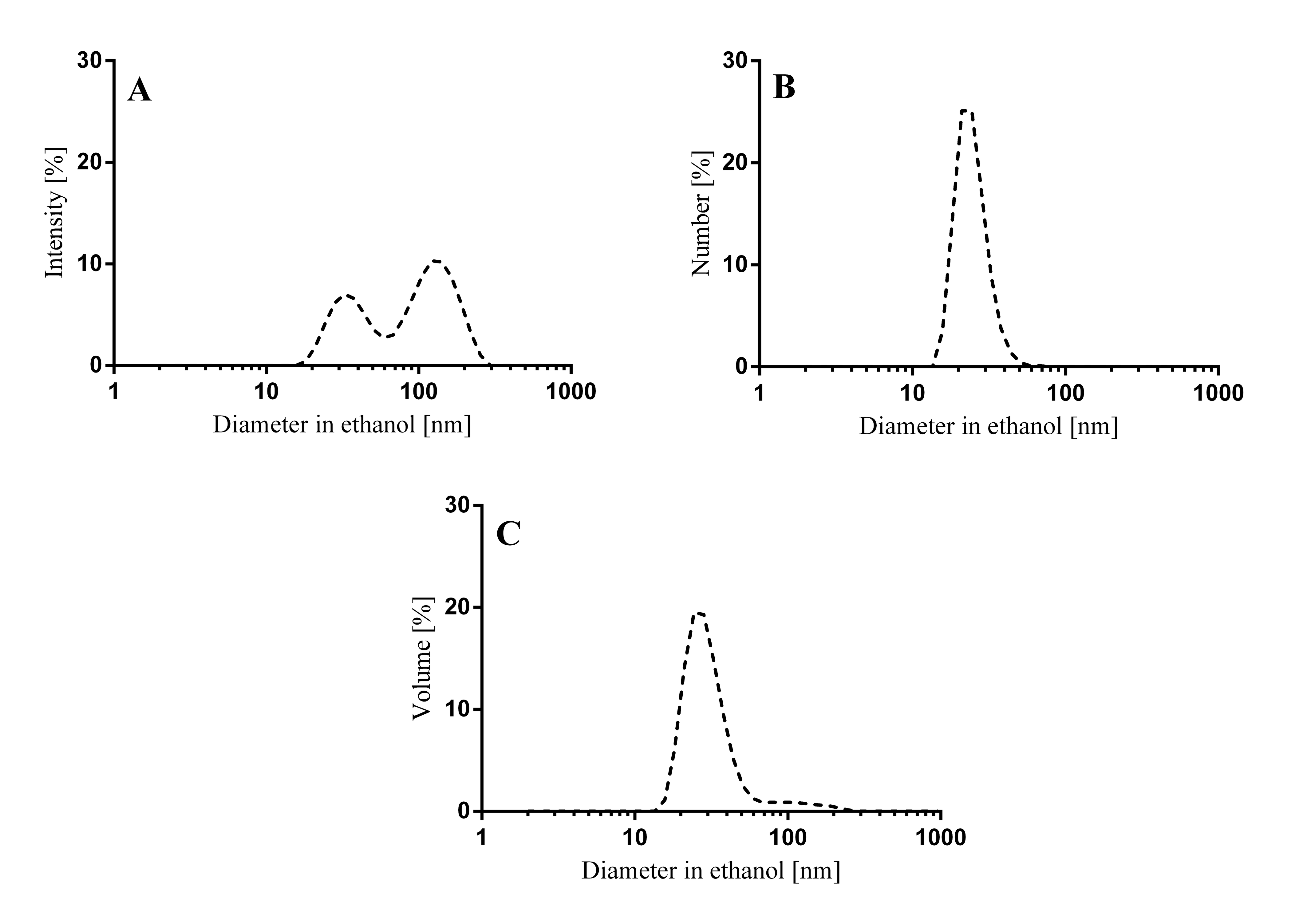

Supplement: S7 Fig — Distributions based on intensity (A), number (B), and volume (C), are shown. This result is based on twenty consecutive measurements of one sample. (TIF) [file pone.0169552.s007.tif]
