# Supplementary material for: Dispersion of Nanoparticles in Different Media Importantly Determines the Composition of Their Protein Corona
Source: PLoS One. 2017 Jan 4;12(1):e0169552. doi: 10.1371/journal.pone.0169552 (PMC5215476; doi:10.1371/journal.pone.0169552)
Supplement: S1 Table — Please note: protein accessions are alphabetically ordered. (PDF) [file pone.0169552.s008.pdf]

Protein accession	Theoretical pI	Molecular mass (Da)	Protein name	Biological process	Molecular function
**A1AT_BOVIN**	5.98	43693.91	α -1-antiproteinase	Protein inhibition	Endopeptidase inhibitor activity
**ALBU_BOVIN**	5.60	66432.96	Serum albumin	Transport	DNA binding, drug binding
**FETUA_BOVIN**	5.10	36353.24	α -2-HS-glycoprotein	Immune system	Endopeptidase inhibitor activity, Promotes endocytosis, Opsonin
**HBBF_BOVIN**	6.51	15859.23	Hemoglobin fetal subunit β	Transport	Oxygen transporter activity
**VTDB_BOVIN**	5.19	51531.70	Vitamin D binding protein	Transport	Vitamin D binding
