# Supplementary material for: Dispersion of Nanoparticles in Different Media Importantly Determines the Composition of Their Protein Corona
Source: PLoS One. 2017 Jan 4;12(1):e0169552. doi: 10.1371/journal.pone.0169552 (PMC5215476; doi:10.1371/journal.pone.0169552)
Supplement: S1 Text — (PDF) [file pone.0169552.s009.pdf]

Formulation for RPMI-1640 Medium ATCC® 30-2001

Inorganic Salts	(g/liter)
$Ca(NO_3)2·4H_2O$	0.10000
$MgSO_4$ (anhydrous)	0.04884
KCl	0.40000
$NaHCO_3$	1.50000
NaCl	6.00000
Na_2HPO_4 (anhydrous)	0.80000

Amino Acids	(g/liter)
L-Arginine (free base)	0.20000
L-Asparagine·H2O	0.05682
L-Aspartic Acid	0.02000
L-Cystine·2HCl	0.06520
L-Glutamic Acid	0.02000
L-Glutamine	0.30000
Glycine	0.01000
L-Histidine (free base)	0.01500
Hydroxy-L-Proline	0.02000
L-Isoleucine	0.05000
L-Leucine	0.05000
L-Lysine·HCl	0.04000
L-Methionine	0.01500
L-Phenylalanine	0.01500
L-Proline	0.02000
L-Serine	0.03000
L-Threonine	0.02000
L-Tryptophan	0.00500
$L\text{-}Tyrosine·2Na·2H_2O$	0.02883
L-Valine	0.02000

Vitamins	(g/liter)
D-Biotin	0.00020
Choline Chloride	0.00300
Folic Acid	0.00100
myo-Inositol	0.03500
Nicotinamide	0.00100
p-Amino Benzoic Acid	0.00100
D-Pantothenic Acid	0.00025
Pyridoxine·HCl	0.00100
Riboflavin	0.00020
Thiamine·HCl	0.00100
Vitamin B-12	0.000005

Other	(g/liter)
D-Glucose	4.50000
Glutathione (reduced)	0.00100
HEPES	2.38300
Phenol Red, Sodium Salt	0.00500
Sodium Pyruvate	0.11000
